# Renal outcomes of STOP-IgAN trial patients in relation to baseline histology (MEST-C scores)

**DOI:** 10.1186/s12882-018-1128-6

**Published:** 2018-11-19

**Authors:** Judith Isabel Schimpf, Till Klein, Christina Fitzner, Frank Eitner, Stefan Porubsky, Ralf-Dieter Hilgers, Jürgen Floege, Hermann-Josef Groene, Thomas Rauen

**Affiliations:** 1 Division of Nephrology and Clinical Immunology, RWTH Aachen University, Pauwelsstr. 30, 52074 Aachen, Germany; 2 Department of Intensive Care, RWTH Aachen University, Aachen, Germany; 3 Department of Medical Statistics, RWTH Aachen University, Aachen, Germany; 4 Bayer AG, Wuppertal, Germany; 5 Cellular and Molecular Pathology, German Cancer Research Center, Heidelberg, Germany; 6 Institute of Pathology, University Medical Centre Mannheim, Mannheim, Germany

**Keywords:** IgA nephropathy, IgAN, MEST-C, Oxford classification, STOP-IgAN

## Abstract

**Background:**

The Oxford classification of IgA nephropathy (IgAN) defines histologic criteria (MEST-C) that provide prognostic information based on the kidney biopsy. There are few data on the predictive impact of this classification in randomized clinical trial settings.

**Methods:**

We performed an exploratory analysis of MEST-C scores in 70 available renal biopsies from 162 randomized STOP-IgAN trial participants and correlated the results with clinical outcomes. Analyses were performed by researchers blinded to the clinical outcome of the patients. Biopsies had been obtained 6.5 to 95 (median 9.4) months prior to randomization.

**Results:**

Mesangial hypercellularity (M1) associated with higher annual eGFR-loss during the 3-year trial (M1: − 5.06 ± 5.17 ml/min/1.73 m^2^, M0: − 0.79 ± 4.50 ml/min/1.73 m^2^, *p* = 0.002). An M0-score additionally showed a weak association with full clinical remission, whereas the percentage of patients losing ≥15 ml/min/1.73 m^2^ over the 3-year trial phase was higher among those scored as M1. Among patients with additional immunosuppression, ESRD occurred more frequently in patients when tubulointerstitial fibrosis (T1/2) was present (T1/2 = 33%, T0 = 0%, *p* = 0.008). In patients receiving supportive care only, ESRD frequencies were similar (T1/2 = 18%, T0 = 7%, *p* = 0.603). At randomization, eGFR was significantly lower when tubulointerstitial fibrosis was present (T1/2: 45.2 ± 15.7 ml/min/1.73 m^2^, T0: 74.6 ± 28.2 ml/min/1.73 m^2^, *p* < 0.0001). Endocapillary hypercellularity (E), and glomerular segmental sclerosis (S) were not associated with any clinical outcome parameter. In the analyzed cohort, patients with glomerular crescents (C1/2 scores) in their biopsies were more likely to develop ESRD during the 3-year trial phase, but this trend was only significant in patients under supportive care.

**Conclusions:**

This secondary analysis of STOP-IgAN biopsies indicates that M1, T1/2 and C1/2 scores associate with worse renal outcomes.

## Background

IgA nephropathy (IgAN) is the most common form of primary glomerulonephritis, presenting with a wide range of clinical features, pathological findings and variable progression of disease [1, 2]. In 2009, the Oxford classification, based on pathological characteristics in IgAN renal biopsies, was introduced to improve individual risk prediction for disease progression. Mesangial hypercellularity (M), endocapillary proliferation (E), segmental glomerulosclerosis or adhesions (S), tubular atrophy and interstitial fibrosis (T) were identified as significant variables predicting renal outcome independent of clinical features [3, 4]. Numerous retrospective analyses aimed to validate these parameters and assessed their predictivity [5–13]. However, these studies did not provide concordant results. Whereas the T-score is consistently accepted as a parameter with high prognostic relevance, the predictive value of M, E and S lesions remains controversial, which might be largely due to differences in patient selection criteria, treatment and outcome measures as well as inter-investigator variability in biopsy assessment [14]. Nonetheless, combination of MEST scores with clinical data at the time of biopsy, i.e. renal function, the degree of proteinuria and arterial hypertension, provided a comparable predictive power as monitoring clinical data over a 2-year period [15]. In 2017, the presence of glomerular crescents (C) was added as a fifth parameter to the revised Oxford classification [16], mainly based on a multicentric analysis of more than 3000 IgAN patients [17].

Despite the continuous improvement in histological characterization and tools to predict disease progression in IgAN patients, optimal therapeutic management on IgAN remains a matter of ongoing debate. There is widely accepted consensus on the essential role of blood pressure and proteinuria control using renin-angiotensin system (RAS) blocking agents. A number of recent randomized clinical trials investigated whether systemic or local immunosuppression on top of comprehensive supportive measures, particularly in patients at risk for a progressive disease course, provides further renal benefits [18–20]. Of note, none of these studies used histologic criteria for trial eligibility and/or patient stratification. Among these trials, STOP-IgAN was the first to evaluate the value of additional systemic immunosuppression in IgAN patients with optimized supportive care. The trial was initiated in 2006, when the Oxford classification was not yet published, and applied a novel, two-phase study design in which 379 patients with biopsy-proven IgAN were enrolled into a 6 months run-in phase of optimizing supportive care measures in accordance with the current KDIGO guidelines [21]. Subsequently, a homogenous high-risk group of 162 patients with persistent proteinuria above 0.75 g/day despite optimized supportive care was randomized to either continue on supportive care (SUP arm) or to receive additional immunosuppression (IMM arm) during a 3-year study phase. Additional immunosuppression induced more full clinical remissions, defined as preserved renal function and proteinuria below 0.2 g/g creatinine at the end of the study phase. However, the overall course of renal function and end-stage renal disease (ESRD) rates were not significantly different between the two arms [20].

Since the Oxford classification of IgAN was introduced in 2009, when our trial had already been initiated, we aimed to collect and re-analyze renal biopsies from randomized STOP-IgAN trial participants using the MEST-C score and to align these criteria with renal outcome data, in particular the two primary end points of the trial, i.e. (1) full remission defined as urinary protein-creatinine ratio < 0.2 g/g and an eGFR-decrease less than 5 ml/min/1.73m^2^ and (2) an eGFR-loss ≥15 ml/min/1.73m^2^ during the trial phase.

## Methods

### Study design

The study protocol and results of STOP-IgAN have been published previously [20, 22]. Briefly, all eligible patients with biopsy-proven IgAN (*n* = 337) entered a 6-months run-in phase with comprehensive optimization of supportive treatment measures. One-hundred-sixty-two patients at high risk for disease progression (i.e. those with a persistent proteinuria > 0.75 g/d, but less than 3.5 g/d, despite optimized supportive care) were then randomized into the following 3-year trial phase and were assigned to either continue on supportive therapy alone or to receive additional immunosuppression.

The Oxford classification of IgAN was introduced in 2009, when the STOP-IgAN trial had already been initiated. Thus, the original trial protocol was amended in 2009 and allowed to retrieve all available original renal biopsies from randomized trial participants. Written informed consent for re-assessment of available kidney biopsies was obtained from all patients included in this secondary substudy.

### Study population

**Fig. 1:**
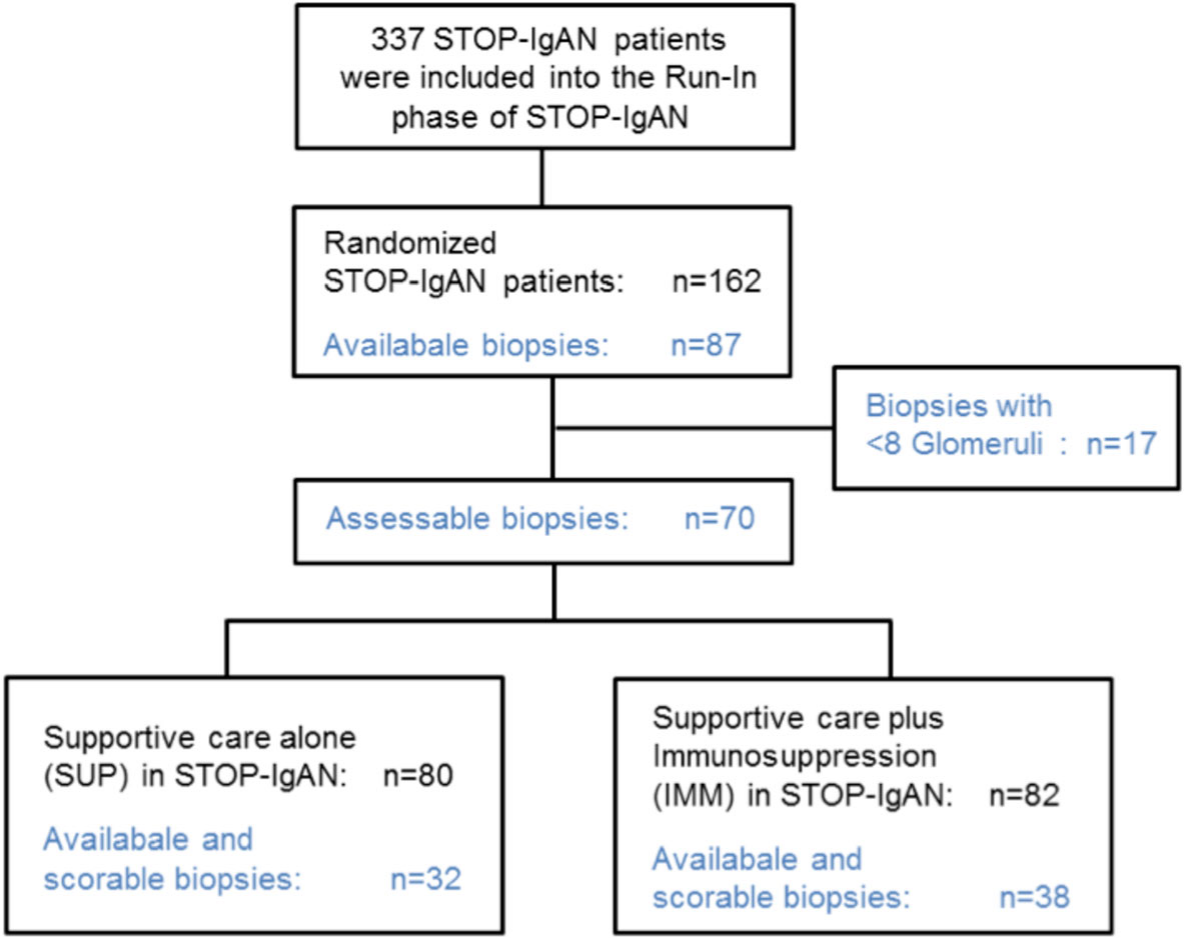
Flowchart of analyzed patients. A total of 337 patients with biopsy-proven IgAN entered the run-in phase of the STOP-IgAN trial during which all patients received supportive care. After 6 months, 162 patients were randomized to either continue on supportive care (*n* = 80) or received additional immunosuppression (*n* = 82). Upon amendment of the initial trial protocol in 2009, we aimed to retrieve the original kidney biopsies from the randomized patients for the current secondary analysis. Eventually, 70 biopsies were collected and could be scored using the MEST-C criteria

This secondary analysis reports data from 87 of the original 162 randomized trial patients. Seventeen biopsies showed less than 8 glomeruli and thus could not be assessed based on the MEST-C criteria. Thirty-two of the remaining 70 patients received supportive care (SUP arm) and 38 patients received immunosuppression in addition to supportive care (IMM arm) during the 3-year study phase (Fig. 1).

### Microscopic analyses

Before enrollment into the STOP-IgAN trial, all patients underwent renal biopsy that was analyzed by a nephropathologist (H.J.G. or one of the pathologists listed in the Acknowledgements). Because of the heterogeneity of the pathology criteria and the high inter-individual examiner bias described in the original publication of the Oxford MEST classification [4], for the present secondary analysis pathology scoring was performed by only one examiner (T.K.), who had been trained and who was subsequently supervised through random control of achieved results obtained from 20 biopsies (i.e. 29% of all biopsies) by two experienced nephropathologists (H.J.G. and S.P.). Concordance rate between MEST-C scoring through T.K. and the one obtained by H.J.G. and S.P. was > 90%. T.K. was blinded to clinical data and previous nephropathologists’ reports. Available renal biopsies were retrieved and analyzed in 2011 according to the current Oxford classification of IgAN using the MEST-C criteria [16]. MEST-C criteria consisted of mesangial hypercellularity (M0: < 50% of glomeruli showing hypercellularity; M1: > 50% of glomeruli showing hypercellularity), endocapillary hypercellularity (E0: absent; E1: present when 3 capillary tubes in two glomeruli showed endocapillary hypercellularity), segmental glomerulosclerosis (S0: absent; S1: present) and tubular atrophy, interstitial fibrosis or interstitial inflammation (T0:< 25%; T1: 25–50%; T2:> 50% of cortical area involved).

**Fig. 2:**
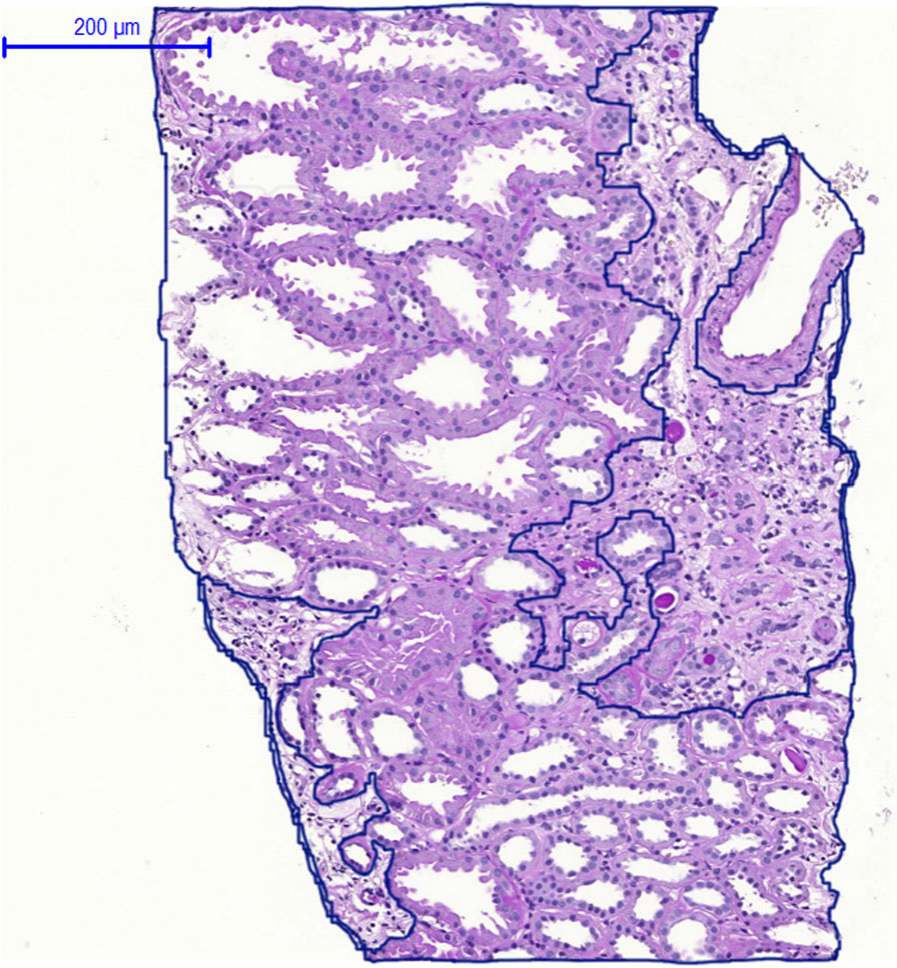
Quantitative morphometry of the T-score in kidney biopsies. In contrast to semiquantitative analyses of tubular atrophy and interstitial fibrosis (T-score) in previous validation studies of the Oxford MEST-C classification, we assessed the T-score of by quantitative morphometry of the tubulointerstitial area using a virtual microscope tool (MIRAX Viewer). The pathologic lesions in the cortical area were encircled and then put in relation to the entire cortical area

The original Oxford classification study as well as the previous validation studies assessed the T-score by visual estimation of the percentage of cortical area involved. Based on these investigations, the T-score is consistently accepted as a histological lesion referring to high prognostic significance [10]. In this study, we used a virtual microscope tool (MIRAX Viewer) to encircle the pathologic lesions in the cortical area and then putting it in relation to the entire cortical area. Thus, we intended to improve the validity of the T-score and to reduce the inter-individual and intra-individual examiner variation to a minimum (Fig. 2).

Crescents were not yet part of the original Oxford classification in 2009 since their prognostic relevance was uncertain at that time, however it was already suggested to also add this information to the biopsy reports. Crescents were then officially introduced in the latest update of the IgA Nephropathy Classification Working Group [16]. In a prescient fashion, the presence of cellular and fibrocellular crescents was also noted and evaluated in our analyses (C0: no crescents, C1: crescents in < 25% of glomeruli, C2: crescents in > 25% of glomeruli).

### Statistical analysis

Data are presented as means ± standard deviations for continuous variables and as counts percentages. Association between MEST-C scores and continuous parameters are analyzed by Satterthwaite t-test. MEST-C scores and binary parameters are analyzed by *Fisher’s exact* tests. The statistical test results are reported by *p*-values. Because of the small sample size, we conducted an explorative statistical analysis only. Consequently, the term “significance” was not used in the statistical confirmatory meaning (by comparison with a significance level). Furthermore, association of selected exploratory variables to renal outcome was evaluated by bivariate analyses (t-test and *Fisher’s exact* test) assuming no confounding factors. Occurrence of the two primary STOP-IgAN endpoints (i.e. achievement of full clinical remission and eGFR-loss ≥15 ml/min/1.73 m^2^) and ESRD was analyzed and visualized by Kaplan-Meier curves using the time of randomization as the starting point. Survival analyses using uni- and multivariate Cox regression (adjusting for GFR and proteinuria at baseline and the treatment arm) were performed as sensitivity analyses to assess the interrelationship between individual MEST scores and these endpoints. For the C-score, Cox regression was not justified since the proportional hazards assumption was not met here. Statistical analyses were performed with SAS (Version 9.4, SAS Institute Inc., Cary, NC, USA).

## Results

### Baseline characteristics of biopsied patients

Among the 162 randomized patients entering the 3-year trial phase, we obtained 87 biopsies. Seventeen biopsies contained fewer than 8 glomeruli and therefore could not be analyzed. Seventy biopsies (43%) fulfilled the required quality criteria proposed for performing the Oxford MEST-C scoring. Of these, 32 were from patients randomized to the SUP arm and 38 to the IMM arm (Fig. 1). Demographic and clinical characteristics of this sub-cohort at the time of enrollment (i.e. at the beginning of the 6-month Run-In phase) are outlined in Table 1 and were similar to the entire study cohort [20]. Time between initial biopsies and trial enrollment ranged between from 6.5 to 95 months (median 9.4 months), however only 6% of patients were biopsied more than 3 years before trial enrollment.

**Table 1:** Patient characteristics at the start of the run-in phase and patients with primary endpoints at the end of the 3-year trial phase

	All (SUP + IMM)	Supportive Care (SUP)	Supportive Care plus Immunosuppression (IMM)
	(*N* = 70)	(*N* = 32)	(*N* = 38)
Patient characteristics:			
Female sex -%	17	13	21
Smoker -%	19	19	18
Age –yr	43.4 ± 13.6	48.2 ± 10.8	39.3 ± 14.5
Body-mass index	28.0 ± 5.0	29.0 ± 4.8	27.3 ± 5.1
Blood pressure- mmHg			
Systolic	131 ± 14	135 ± 16	127 ± 12
Diastolic	82 ± 11	86 ± 11	79 ± 10
Serum creatinine -mg/dl	1.4 ± 0,5	1.5 ± 0.5	1.3 ± 0.5
eGFR-ml/min/1.73m^2^	67 ± 28	61 ± 25	73 ± 30
Creatinine clearance - ml/min	82 ± 34 (*N* = 60)	80 ± 30 (*N* = 28)	84 ± 37 (N = 32)
Daily urinary protein excretion - g/day	2.3 ± 1.3	2.4 ± 1.3	2.2 ± 1.3
Urinary protein-creatinine ratio - g/g	1.4 ± 0.8 (N = 60)	1.4 ± 0.8 (N = 28)	1.4 ± 0.8 (*N* = 32)
Cholesterol - mg/dl	210 ± 44 (*N* = 67)	208 ± 37 (*N* = 29)	211 ± 48(N = 38)
Primary endpoints:			
Full clinical remission^a^	8	2	6
eGFR-loss ≥15 ml/min	23	11	12
ESRD^b^	8	4	4

^a^urinary protein-creatinine ratio < 0.2 g/g and an eGFR decrease of < 5 ml/min/1.73m^2^

^b^end-stage renal disease

### Pathology score distribution

Among the randomized patients followed through the 3-year trial phase, 26% had diffuse mesangial hypercellularity (M1), 17% endocapillary hypercellularity (E1), 91% segmental glomerulosclerosis (S1) and 41% tubular atrophy and interstitial fibrosis (T1/T2). Consistent with the VALIGA cohort [6] T2 lesions were rare (only in 4% of analyzed patients), therefore T1 and T2 lesions were combined to facilitate subsequent statistical analyses. The same procedure was applied to the glomerular crescent scores C1 and C2 (only 7% had a C2-score), resulting in 31% of biopsies showing crescents (C1/C2) (Table 2).

**Table 2:** MEST-C score distribution in STOP-IgAN patients

	All (SUP + IMM)	Supportive Care (SUP)	Supportive Care plus Immunosuppression (IMM)
	(*N* = 70)	(*N* = 32)	(*N* = 38)
Mesangial hypercellularity			
M0	52 (74%)	24 (75%)	28 (74%)
M1	18 (26%)	8 (25%)	10 (26%)
Endocapillary hypercellularity			
E0	58 (83%)	27 (84%)	31 (82%)
E1	12 (17%)	5 (16%)	7 (18%)
Segmental glomeruloscerlosis			
S0	6 (9%)	3 (9%)	3 (8%)
S1	64 (91%)	29 (91%)	35 (92%)
Tubular atrophy/Interstitial fibrosis			
T0	41 (59%)	15 (47%)	26 (68%)
T1	26 (37%)	17 (53%)	9 (24%)
T2	3 (4%)	0 (0%)	3 (8%)
Crescents			
C0	48 (69%)	24 (75%)	24 (63%)
C1	17 (24%)	7 (22%)	10 (26%)
C2	5 (7%)	1 (3%)	4 (11%)

Within the analyzed sub-cohort baseline eGFR was significantly lower in patients with T1/T2 scores as compared to those with a T0-score (T1/2: 45 ± 16 ml/min/1.73 m^2^ vs. T0: 75 ± 28 ml/min/1.73 m^2^, *p* < 0.0001; Table 3). Mean baseline proteinuria levels did not differ between the T0 and the T1/2 group. Moreover, no obvious differences in eGFR, occurence of microhematuria and proteinuria were observed in the M, S, E and C categories.

**Table 3:** Association between MEST-C score and baseline eGFR in STOP-IgAN patients

	Mean eGFR^a^^,^^b^	*p-value*
Mesangial hypercellularity		0.674
M0	62 ± 26	
M1	65 ± 34	
Endocapillary hypercellularity		0.308
E0	64 ± 27	
E1	53 ± 34	
Segmental glomerulosclerosis		0.422
S0	74 ± 34	
S1	61 ± 27	
Tubular atrophy/Interstitial fibrosis		< 0.0001
T0	75 ± 28	
T1/2	45 ± 16	
Crescents		0.815
C0	62 ± 29	
C1/2	64 ± 25	

^a^end of the 6-month run-in phase

^b^ ml/min/1.73m^2^

### Association between MEST-C scores and clinical outcome data

Patients with an M1-score had fewer full clinical remissions (M1: 0%vs. M0: 17%, *p* = 0.099; Table 4*)* and a higher incidence of eGFR-loss ≥15 ml/min/1.73m^2^ during the trial phase (M1: 50%vs. M0: 27%, *p* = 0.092), yet these trends did not reach the level of statistical significance. There were no differences in remission rates between individuals in the different study arms, neither in patients under supportive care (M1: 0%, M0: 9%, *p* = 1.0, Table 5), nor in patients under additional immunosuppression (M1: 0%, M0: 23%, *p* = 0.304, Table 5). Mean annual GFR loss was significantly higher for patients in the M1 group than for those in the M0 group (M0: − 0.79 ± 4.50 ml/min/1.73 m^2^ vs. M1–5.06 ± 5.17 ml/min/1.73 m^2^, *p* = 0.002).

**Table 4:** Association between M-, T- and C- scores and clinical outcome in STOP-IgAN patients (available cases analysis)

	M0 events/ total	M1 events/ total	*p-value*	T0 events/ total	T1/2 events/ total	*p-value*	C0 events/ total	C1/2 events/ total	*p-value*
Full clinical remission^a^	8/48 (17%)	0/17 (0%)	*0.099*	6/39 (15%)	2/26 (8%)	*0.460*	4/45 (9%)	4/20 (20%)	*0.238*
GFR-loss ≥15 ml/min	14/51 (27%)	9/18 (50%)	*0.092*	11/40 (28%)	12/29 (41%)	*0.302*	16/47 (34%)	7/22 (32%)	*1.000*
ESRD^b^	7/51 (14%)	1/18 (6%)	*0.671*	1/40 (3%)	7/29 (24%)	*0.008*	4/47 (9%)	4/22 (18%)	*0.255*
Disappearance of microhematuria	11/34 (32%)	3/15 (20%)	*n.d.* ^*c*^	10/29 (35%)	4/20 (20%)	*n.d.* ^*c*^	10/34 (29%)	4/15 (27%)	*1.000*
Absolute annual GFR change (ml/min/1.73m^2^)	−0.79 ± 4.50	−5.06 ± 5.17	*0.002*	−2.05 ± 5.40	−2.01 ± 4.54	*0.362*	− 2.66 ± 5.02	−0.82 ± 5.02	*0.131*

^a^urinary protein-creatinine ratio < 0.2 g/g and an eGFR decrease of < 5 ml/min/1.73m^2^

^b^end-stage renal disease

^c^not determined (due to the low total numbers of available data points in this category, *n* = 49)

**Table 5:** Association between M-, T- and C- scores and clinical outcome in the two treatment arms of the STOP-IgAN trial (available cases analysis)

		M0 events/ total	M1 events/ total	T0 events/ total	T1/2 events/ total	C0 events/ total	C1/2 events/ total
Full clinical remission^a^	SUP^c^	2/22 (9%)	0/8 (0%)	2/15 (13%)	0/15 (0%)	1/23 (4%)	1/7 (14%)
	IMM^d^	6/26 (23%)	0/9 (0%)	4/24 (17%)	2/11 (18%)	3/22 (14%)	3/13 (23%)
GFR-loss ≥15 ml/min	SUP	7/24 (29%)	4/8 (50%)	4/15 (27%)	7/17 (41%)	6/24 (25%)	5/8 (63%)
	IMM	7/27 (26%)	5/10 (50%)	7/25 (28%)	5/12 (42%)	10/23 (43%)	2/14 (15%)
ESRD^b^	SUP	3/24 (13%)	1/8 (13%)	1/15 (7%)	3/17 (18%)	1/24 (4%)	3/8 (38%)
	IMM	4/27 (15%)	0/10 (0%)	0/25 (0%)	4/12 (33%)	3/23 (13%)	1/14 (7%)

^a^urinary protein-creatinine ratio < 0.2 g/g and an eGFR decrease of < 5 ml/min/1.73m^2^

^b^end-stage renal disease

^c^patients under supportive treatment during the 3-year trial phase

^d^patients under additional immunosuppression during the 3-year trial phase

In our cohort, T1/2-scores did not correlate with full clinical remission or eGFR-loss rates during the trial phase, however, T1/2 was significantly associated with ESRD development (T1/2: 24% vs. T0: 3%, *p* = 0.008). This association was exclusively observed in patients under additional immunosuppression (T1/2: 44% vs. T0: 0%, p = 0.008), but not among those under supportive treatment (T1/2: 18% vs. T0: 7%, *p* = 0.603). Mean baseline eGFR in the four patients under additional immunosuppression with an T1/2 score who developed ESRD was 47 ± 15 ml/min/1.73 m^2^ and among the three patients with an T1/2 in the supportive care group 51 ± 8 ml/min/1.73 m^2^. Urinary protein-creatinine ratios did not differ between these subcohorts (1,7 ± 0,8 g/g for both).

**Fig. 3:**
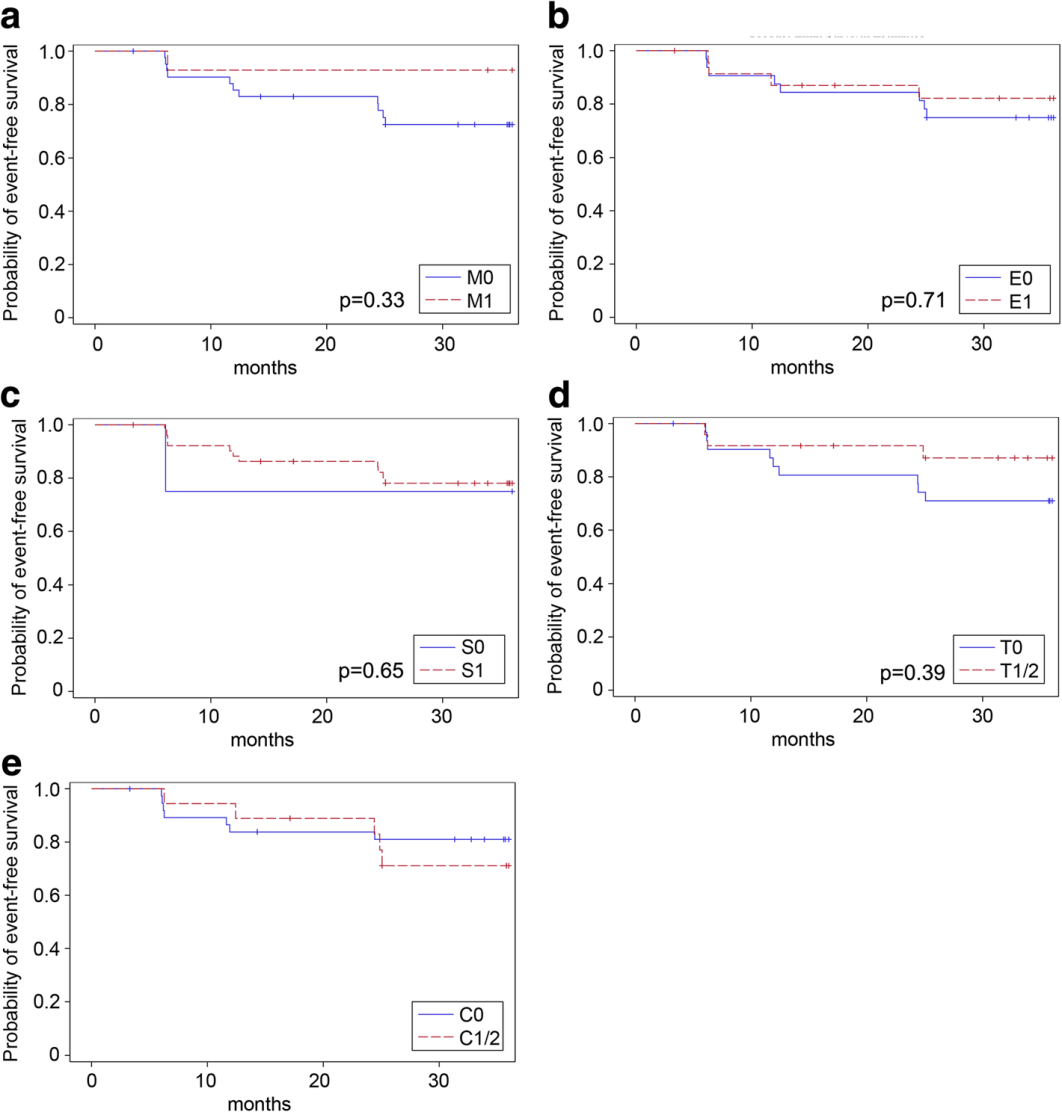
Kaplan-Meier curves for the event “*full clinical remission*” based on the M-score (**a**), E-score (**b**), S- score(**c**), T-score (**d**) and C-score (**e**). Full clinical remission was defined as urinary protein-creatinine ratio < 0.2 g/g and an eGFR decrease of < 5 ml/min/1.73m^2^. Univariate Cox regression yielded the following *p*-values: M-score: *p* = 0.41; E-score: *p* = 0.46; S-score: *p* = 0.38; T-score: *p* = 0.39. Multivariate Cox regression adjusting for GFR and proteinuria at baseline and the treatment arm yielded the following *p*-values (also given in the figure): M-score: *p* = 0.33; E-score: *p* = 0.71; S-score: *p* = 0.65; T-score: *p* = 0.39). For the C-score, the proportional hazard assumption was not met

**Fig. 4:**
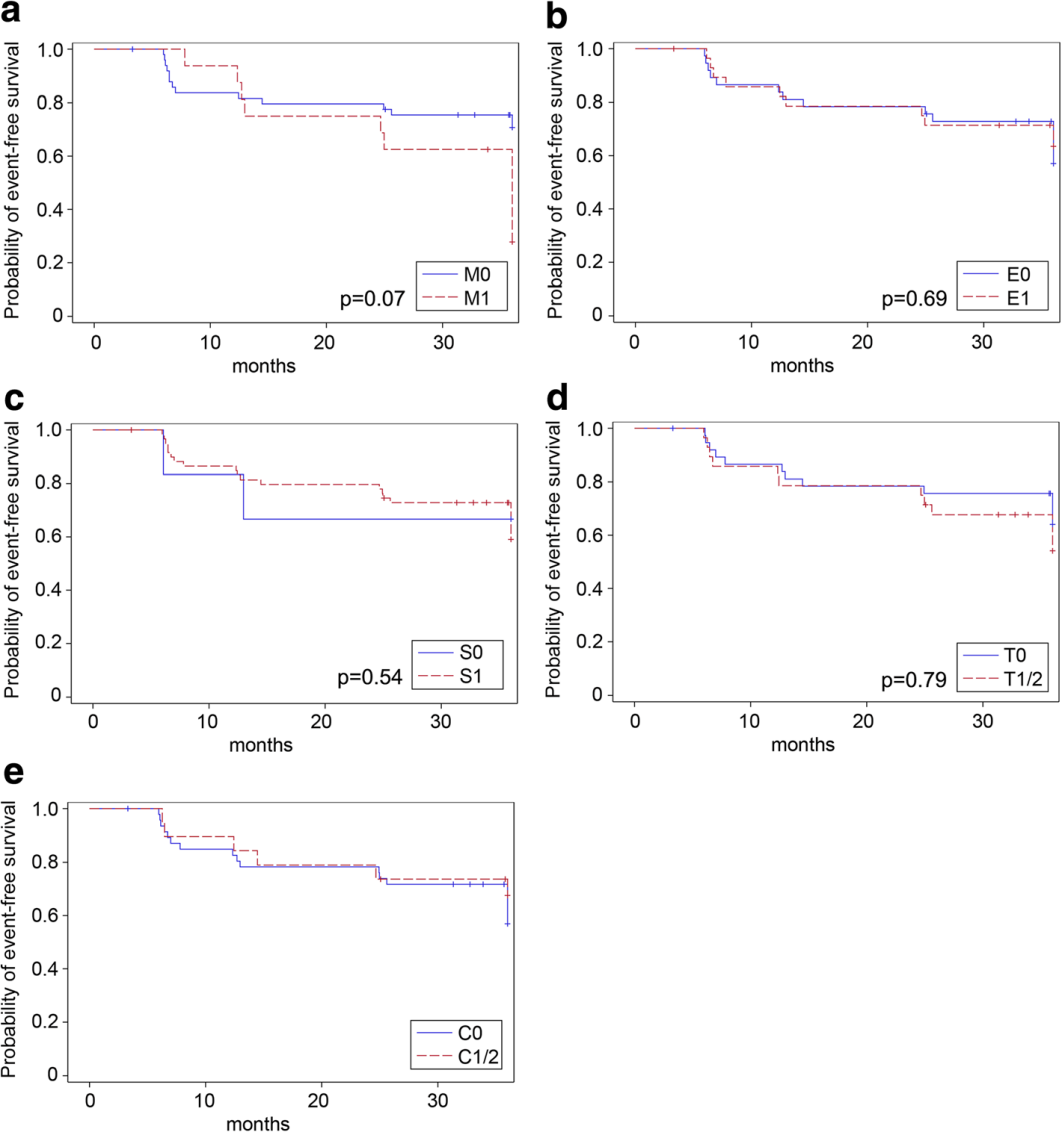
Kaplan-Meier curves for the event “*eGFR-loss ≥15 ml/min/1.73 m*^*2*^” based on the M-score (**a**), E-score (**b**), S-score (**c**), T-score (**d**) and C-score (**e**). Univariate Cox regression yielded the following *p*-values: M-score: *p* = 0.08; E-score: *p* = 0.41; S-score: *p* = 0.91; T-score: *p* = 0.15. Multivariate Cox regression adjusting for GFR and proteinuria at baseline and the treatment arm yielded the following *p*-values (also given in the figure): M-score: *p* = 0.07; E-score: *p* = 0.69; S-score: *p* = 0.54; T-score: *p* = 0.79). For the C-score, the proportional hazard assumption was not met

**Fig. 5:**
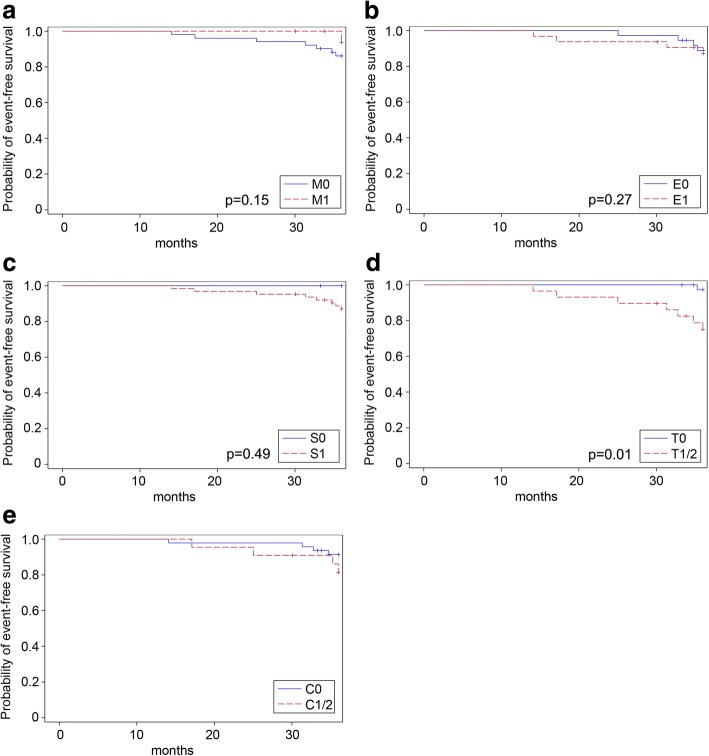
Kaplan-Meier curves for the event “ESRD development” based on the M-score (**a**), E-score (**b**), S-Sscore (**c**), T-score (**d**) and C-score (**e**). Univariate Cox regression yielded the following *p*-values: M-score: *p* = 0.32; E-score: *p* = 0.96; S-score: *p* = 0.90; T-score: *p* = 0.02. Multivariate Cox regression adjusting for GFR and proteinuria at baseline and the treatment arm yielded the following *p*-values (also given in the figure): M-score: *p* = 0.15; E-score: *p* = 0.27; S-score: *p* = 0.49; T-score: *p* = 0.01). For the C-score, the proportional hazard assumption was not met

ESRD occurred more frequently in patients with glomerular crescents (C1/C2) than in patients with a C0-score (C1/2: 18% vs. C0: 9%, *p* = 0.255), whereas this trend was only significant in patients receiving supportive therapy (C1/2: 38% vs. C0: 4%, *p* = 0.039, Table 5). Accordingly, eGFR-loss rates of at least 15 ml/min/1.73m^2^ did not correlate with crescents in patients receiving supportive therapy (C1/2: 63% vs. C0: 25%, *p* = 0.088) nor in those receiving additional immunosuppression (C1/2: 15% vs. C0: 43%, *p* = 0.084, Table 5). Occurrence of primary trial endpoints (full clinical remission and eGFR-loss ≥15 ml/min/1.73m^2^) and ESRD over the 3-year trial phase in the individual MEST-C subgroups was also visualized in Kaplan-Meier curves (Figs. 3, 4 and 5). Sensitivity analyses including uni- and multivariate Cox regression analyses entirely confirmed the previous trends for the MEST criteria as the only significant association in our cohort was observed for the T-score and ESRD development (*p* = 0.02 in the univariate Cox regression model; *p* = 0.01 in the multivariate Cox regression adjusting for GFR and proteinuria at baseline and the treatment arm).

Endocapillary hypercellularity (E) and segmental glomerular sclerosis (S) did not correlate with any of the other analyzed clinical outcome parameters. Disappearance of microhematuria did not correlate with any of the MEST-C criteria.

In our cohort, thrombotic microangiopathy (TMA) lesions were screened in randomly selected biopsies and were observed in only 2–4% of biopsies at maximum.

## Discussion

All randomized clinical trials (RCTs) that evaluated therapeutic strategies in IgAN patients, of course required a renal biopsy as an eligibility criterion to confirm the diagnosis of IgAN. However, none of these trials, including the most recent ones [18–20], applied pre-defined histological features such as the MEST-C parameters for patient selection or stratification nor did these trials prospectively analyze renal outcomes in individual histological subgroups. In this regard, STOP-IgAN is no exception which is not surprising since the first version of the Oxford classification was published when STOP-IgAN was already recruiting patients [3, 4].

Here, we present an exploratory analysis from a representative STOP-IgAN subcohort [20] that includes 43% of randomized patients with available and sufficient biopsies that were scored based on the current MEST-C classification.

Baseline eGFR was significantly lower in patients with T1/2 scores as compared to those with T0. This is not surprising since tubular atrophy and interstitial fibrosis are hallmark features of irreversible kidney damage and markers of advanced stages of renal disease regardless of the underlying pathology. Our data confirm older studies that tubulointerstitial damage in IgAN exhibits a very close association with renal function [23]. Retrospective data from the European VALIGA cohort that included more than 1100 IgAN patients suggested that the T-score was consistently predictive for poor renal outcomes, also in patients with a baseline GFR below 30 ml/min/1.73m^2^ [6]. Notably, patients with such low renal function at enrollment had been excluded in STOP-IgAN and other randomized controlled trials. A large cohort of Korean IgAN patients also exhibited a significant correlation between T-scores and eGFR at the time of the biopsy [8]. In line with this, STOP-IgAN patients with biopsies showing T1/2 scores had a lower mean baseline eGFR as compared to the the whole study cohort and were more likely to progress to end-stage renal disease (ESRD). Subgroup-analyses showed that T1/2-scores were only predictive for ESRD among patients who received additional immunosuppression and not in those under supportive care albeit only eight patients in the present subcohort (11%) developed ESRD (four patients in each treatment arm). Although the present secondary analysis only included 70 IgAN patients, it is worth noting that unlike many preceding clinical trials, all STOP-IgAN patients received RAS-blocking agents in a standardized fashion [20], i.e. dose titration based on proteinuria and blood pressure levels. To our knowledge, the current analysis from the STOP-IgAN cohort is the first one suggesting a potential interaction between tubular atrophy/interstitial fibrosis and immunosuppression. Lv et al. had pooled renal outcome data from 16 retrospective cohort studies comprising more than 3800 IgAN patients and found that the presence of a T1/2 score heralded an increased risk for ESRD development (HR 3.2; 95%-CI 1.8–5.6; *p* < 0.001) [10]. In general, T-scoring has proven to be a valuable predictor in nearly all validation studies [14].

In contrast, the predictive impact of endocapillary hypercellularity (E-score) is a matter of ongoing debate. In the above cited VALIGA cohort, the E-score was not predictive in the entire population or various subgroups [6]. Similar results were reported in the meta-analysis of Lv et al. [10]. However, other studies suggested that endocapillary lesions might respond to immunosuppressive therapy: a subgroup analysis from the original Oxford classification study revealed that the annual GFR-loss was significantly higher in patients scored as E1 as compared to those with E0, however only in patients without immunosuppression and not among those receiving immunosuppression [3]. Along these lines, a recently published, single-center study confirmed an E1-score as an independent predictor for ESRD in patients who did not receive immunosuppression [24]. In contrast to these studies, in STOP IgAN patients E-scoring did not predict any measured outcome.

Mesangial hypercellularity (M-score) is considered a very sensitive pathology marker in predicting disease progression [6, 25]. In accordance with the VALIGA cohort [6], STOP-IgAN patients with an M1-score had a significantly higher annual loss of renal function than patients scored as M0. However, M-scoring did not show a significant association with the percentage of patients losing > 15 ml/min/1.73 m^2^ of GFR and ESRD occurrence. The reason for this might relate to the limited observation time of 3 years only and the overall low number of ESRD events in the analyzed subcohort. Furthermore, the predictive value of M-scoring might be abandoned if patients receive immunosuppressive therapy [6] and indeed in our subgroup of immunosuppressed STOP-IgAN patients (IMM arm), the M-score was not validated as an independent risk factor. Previous cohort analyses suggest that in IgAN patients at more advanced disease stages M-scoring is no longer predictive [5, 7]. Further studies are needed to evaluate the relationship between supportive and/or immunosuppressive therapy and mesangial hypercellularity as a disease predictor.

Crescents have been introduced as the C-score only very recently in the revised Oxford classification [16]. This was based on several observations from smaller studies and a large IgAN cohort pooled from four previous analyses [17]. The latter found that patients with glomeruli containing crescents had a worse renal outcome than those without crescents. In the STOP-IgAN subcohort, analyzed patients displaying glomerular crescents in their biopsies were more likely to lose at least 15 ml/min/1.73m^2^ of GFR or to develop ESRD during the 3-year trial phase. This trend was only significant among patients under supportive care and not in those under additional immunosuppression. This might indicate that the cellular proliferative component in the extracapillary space is responsive to immunosuppressive therapy [17, 26]. Whether in fact immunosuppression has beneficial effects on active crescentic lesions and results in subsequent GFR improvement is an intriguing hypothesis that needs to be evaluated in future studies.

We did not find evidence for frequent thrombotic microangiopathy (TMA) lesions in the biopsies from our STOP-IgAN subcohort. At maximum, we observed 2–4% TMA lesions in our patients contrasting data from a French single-center study reporting > 50% of such lesions [27], however our findings are consistent with data from other cohorts [28].

Compared to numerous preceding studies that aimed to validate the original Oxford classification, our study has several strengths. First, we applied a novel morphometric tool to more reliably quantify the degree of tubulointerstitial damage. The original Oxford Classification study as well as the subsequent validation studies only assessed the T-score by rough visual estimation of the involved cortical area [3, 4, 16]. Given its high prognostic significance on renal outcome, our approach might help to improve the validity of the T-score and minimize the inter- and intra-individual examiner variation. Furthermore, to our knowledge, this is the first validation study assessing all five parameters of the updated Oxford classification of IgAN including the presence of crescents in a prospective clinical trial.

Limitations of this secondary analysis include its *post-hoc* character and the variable intervals between time point of kidney biopsy and study enrollment (between 6.5 and 95 months), however only 6% of the biopsies were performed more than 3 years before trial enrollment. It might well be that with progressing disease course between kidney biopsy and trial enrollment, active renal lesions such as E- or C-lesions might transform into more chronic pathological manifestations. Thus, our study bares a certain “observational gap” between the actual biopsy and the time of trial inclusion. However, given the overall very slow annual decline of renal function, even in IgAN patients under supportive therapy only (approx. –1,5 ml/min/1.73m^2^ per year), we consider this relatively short median time span of 9.4 months not relevant with respect to the chosen renal outcome parameters. Since the original Oxford classification was published in 2009, when STOP-IgAN was already recruiting patients, these histopathological characteristics were not “state of the art” at the time of enrollment and had to be obtained *ex post*. However, the histological analyses described here were performed blindly with regard to clinical trial data. Nowadays, IgAN kidney biopsies are reported based on the updated Oxford criteria in a standardized fashion [16]. The number of kidney biopsies to which the current MEST-C criteria were applicable was limited to 43% of randomized STOP-IgAN patients. Unfortunately, it was not feasible to obtain biopsies from all randomized patients, in part, because in the STOP-IgAN trial protocol, original biopsies were not requested to be delivered to the central trial coordinator. However, with regard to baseline renal function, proteinuria and other major patient characteristics the analyzed subcohort was representative for the entire STOP-IgAN population [20]. Nevertheless, given this sample size our *post-hoc* analysis was not sufficiently powered to determine interrelationships between histopathological scores and treatment effects. Moreover, because of the small sample size, we conducted an explorative statistical analysis only.

## Conclusions

We applied the current Oxford classification to 70 randomized STOP-IgAN patients, either receiving immunosuppressive or supportive therapy only. Mesangial hypercellularity associated with a more rapid annual decline of eGFR, whereas the degree of tubular atrophy and interstitial fibrosis was predictive for ESRD, particularly in patients under immunosuppressive therapy. Since approximately one third of IgAN patients progresses to ESRD over 20–40 years [29], it is of outmost importance to identify patients early who are at risk for a progressive disease course. M1- as well as T1/2 and C1/C2 scores in the kidney biopsy might serve as valuable parameters to identify such high-risk candidates.

## Abbreviations

eGFR: Estimated glomerular filtration rate
ESRD: End-stage renal disease
IgAN: Immunoglobulin A nephropathy
IMM arm: Patients under additional immunosuppression on top of optimized supportive care in the STOP-IgAN trial
KDIGO: Kidney Disease Improving Global Outcomes
MEST-C: (M)esangial hypercellularity, (E)ndocapillary hypercellularity, (S)egmental glomerulosclerosis, (T)ubular atrophy and interstitial fibrosis, (C)rescents
RAS: Renin-angiotensin system
SUP arm: Patients under optimized supportive care in the STOP-IgAN trial
TMA: Thrombotic microangiopathy
VALIGA study: European VALidation Study of the Oxford Classification of IGA Nephropathy
